# Mortality rate among HIV-positive children on ART in Northwest Ethiopia: a historical cohort study

**DOI:** 10.1186/s12889-020-09418-6

**Published:** 2020-08-27

**Authors:** Animut Alebel, Eshetu Haileselassie Engeda, Mengistu Mekonnen Kelkay, Pammla Petrucka, Getiye Dejenu Kibret, Fasil Wagnew, Getnet Asmare, Zebenay Workneh Bitew, Daniel Bekele Ketema, Getnet Gedif, Belisty Temesgen, Yitbarek Tenaw Hibstie, Mamaru Wubale Melkamu, Setegn Eshetie

**Affiliations:** 1 College of Health Science, Debre Markos University, P.O. Box 269, Debre Markos, Ethiopia; 2 Australian Centre for Public and Population Health Research, School of Public Health, Faculty of Health, University of Technology Sydney, Ultimo, NSW Australia; 3 College of Medicine and Health Sciences, University of Gondar, Gondar, Ethiopia; 4 College of Nursing, University of Saskatchewan, Saskatoon, Canada; 5 School of Life Sciences and Bioengineering, Nelson Mandela African Institute of Science and Technology, Arusha, Tanzania; 6 Debre Tabor University, College of Health Sciences, Debre Tabor, Ethiopia; 7 Department of Nursing, St. Paul’s Hospital Millennium Medical College, Addis Ababa, Ethiopia; 8 Debre Markos Referral Hospital, Debre Markos, Ethiopia

**Keywords:** Amhara region, Ethiopia, ART, HIV-positive, Mortality

## Abstract

**Background:**

Though highly active antiretroviral therapy (HAART) has been available for more than a decade in Ethiopia, information regarding mortality rates of human immunodeficiency virus (HIV)-positive children after antiretroviral therapy antiretroviral therapy (ART) initiation is very scarce. Thus, this study intends to determine the predictors of mortality among HIV-positive children receiving ART in Amhara Region.

**Methods:**

A multicenter facility-based historical cohort study was conducted in 538 HIV-positive children on ART from January 2012 to February 2017. We employed a standardized data extraction tool, adapted from ART entry and follow-up forms. Descriptive analyses were summarized using the Kaplan-Meier survival curve and log rank test. Then, the Cox-proportional hazard regression model was employed to estimate the hazard of death up to five-years after ART initiation. Variables with *p*-values ≤0.25 in bivariable analysis were candidates to the multivariable analysis. Finally, variables with p-values < 0.05 were considered as significant variables.

**Results:**

The cohort contributed a total follow-up time of 14,600 child-months, with an overall mortality rate of 3.2 (95% CI: 2.3, 4.3) per 100 child-years. This study also indicated that HIV-infected children presenting with opportunistic infections (OIs) (AHR: 2.5**,** 95% CI: 1.04, 5.9), anemia (AHR: 3.1, 95% CI: 1.4, 6.7), severe immunodeficiency (AHR: 4.4, 95% CI: 1.7, 11.7), severe stunting (AHR: 3.3, 95% CI: 1.4, 8.0), severe wasting (AHR: 3.1, 95% CI: 1.3, 7.3), and advanced disease staging (III and IV) (AHR: 3.0, 95% CI: 1.2, 7.1) were at higher risk of mortality.

**Conclusion:**

A higher rate of mortality was observed in our study as compared to previous Ethiopian studies. HIV-positive children presenting with anemia, OIs, severe immunodeficiency, advanced disease staging (III and IV), severe stunting, and severe wasting were at higher risk of mortality.

## Background

Human immunodeficiency virus (HIV) remains a serious public health concern worldwide, but low and middle-income countries (LMICs) are the most affected. In 2018, approximately 37.9 million people were living with HIV worldwide, of whom 1.8 million were children (age < 15 years) [1]. Sub-Saharan Africa (SSA) is a disproportionally affected region contributing to approximately 91% of HIV-positive children in 2012 [2]. Globally, Acquired Immune Deficiency Syndrome (AIDS) remains the leading cause of mortality among children, resulting in 190,000 children deaths in 2013 [3]. In Ethiopia, 109,133 children are living with HIV and approximately 2420 are newly infected with HIV annually [4].

The use of anti-retroviral therapy (ART) has been effective in reducing mortality markedly among infected children and adolescents [5]. In this regard, the World Health Organization (WHO) exceeded its target of 15 million ART users by the end of 2015. Likewise, the Ethiopian government launched cost-based ART in 2003 and cost-free ART in 2005, delivered as part of comprehensive HIV/AIDS care [6]. As a result, ART coverage among children in Ethiopia has increased from 9% in 2013 to 58% in 2018 [7]. According to the recent Ethiopian Demographic and Health Survey (EDHS, 2016), child mortality in Ethiopia was reported as 67 per 1, 000 live births. In our study area (Amhara Region), child mortality rate was reported as 85 per 1, 000 live births [8].

Despite different interventions undertaken by the Ethiopian government, the mortality rate among HIV infected children remains higher than the general pediatric population. Previous studies done in Ethiopia reported that the mortality rate in HIV-positive children receiving ART ranged from 1.4 deaths per 100 child-years of observation [9] to 4 deaths per 100 child-years of observation [5]. These studies also reported that many factors significantly increased the risk of mortality in this population. For example, baseline anemia (Hgb < 10 g/dL) [5, 9], low CD4 count [5], advanced WHO clinical disease staging (III & IV) [5, 9], poor ART drug adherence [10], and malnutrition [11] were reported as factors significantly increasing risk of mortality in HIV-positive children receiving ART.

Obtusely, well-organized and up-to-date information concerning mortality rate of HIV-positive children after ART initiation is highly recommended to provide quality care to HIV-positive children. However, although Amhara region has a particularly high child mortality rate, a minimal number of studies have been conducted in this area. Further, even though a few studies have been conducted in Ethiopia, these studies have failed to consider important predictors, such as malnutrition and developmental status of the children, which significantly affect the mortality rate of HIV-positive children [11, 12]. Thus, this study was conducted to address this knowledge and evidentiary gap by identifying the predictors of mortality among HIV-positive children receiving ART in Amhara Region referral hospitals, Northwest Ethiopia.

## Methods

### Study settings and period

This study was done in three purposely selected Amhara Region Referral Hospitals between January 2012 and February 2017. The three selected hospitals were Debre Markos Referral Hospital, Felege Hiwot Comprehensive Specialized Hospital, and University of Gondar Comprehensive Specialized Hospital, with respective locations of 299 km, 578 km, and 725 km from Addis Ababa, the capital city of Ethiopia. These hospitals were selected because they provide ART follow-up and care services for a large proportion of HIV-positive patients in the region. Together three hospitals provide inpatient and outpatient services for more than 15 million people living in the Amhara Region and neighboring regions. In addition to other medical services, all the three hospitals provide chronic HIV care (ART) for approximately 1071 children in their catchment areas.

### Study design and participants

A multicenter facility-based historical cohort study was undertaken. All children living with HIV receiving ART in all Amhara Region Referral Hospitals were our target population. All randomly selected HIV-positive children who took ART medication for a minimum of 1 month during the follow-up period in the study hospitals were included. Excluded participants were those with incomplete baseline variables.

### Sample size and sampling technique

The minimum required sample size was estimated based on the following variables: severe wasting, severe underweight, CD4 counts or percentage below the threshold, anemia (Hgb < 10 g/dL), and WHO clinical stage III and IV. However, the maximum calculated sample size was obtained from CD4 cell count or percentage below the threshold and was taken as our final sample size. Our final sample size was calculated based on the following parameters: P1: percent of exposed (CD4 count or % below the threshold) with outcome (11.6%), P2: percent among non-exposed (CD4 count or % above the threshold) with outcome (4.4%), 95% CI, **Z**_**β**_ = 80% and r = 1: 1 ratio. The final sample size after adding a10% chart incompleteness rate was 553. The parameters were taken from a previous study conducted at Bahir Dar Referral Hospital [5].

**Fig. 1:**
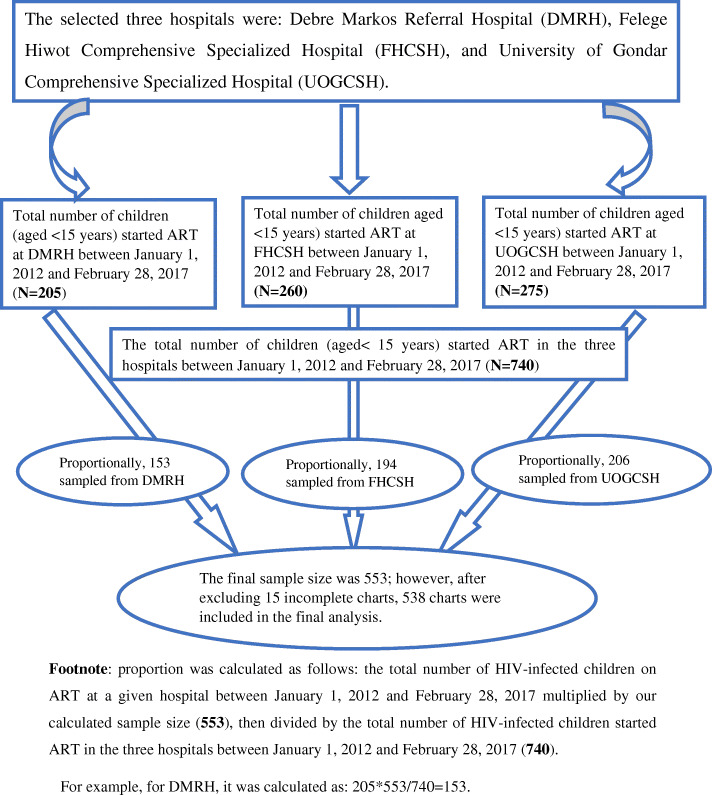
Schematic presentation of sampling procedure to assess the predictors among HIV-infected children on ART in Amhara Region Referral Hospitals from January 1, 2012 to February 28, 2017. Footnote: proportion was calculated as follows: the total number of HIV-infected children on ART at a given hospital between January 1, 2012 and February 28, 2017 multiplied by our calculated sample size (553), then divided by the total number of HIV-infected children started ART in the three hospitals between January 1, 2012 and February 28, 2017 (740). For example, for DMRH, it was calculated as: 205*553/740 = 153

First, the medical charts of HIV-positive children started on ART (740) prior to the follow-up period at the three selected hospitals were sorted. Next, 553 children’s medical records were selected using a simple random sampling technique. Lastly, data across 5 years were extracted from each enrolled child’s record (Fig. 1).

### Data extraction procedures

After reviewing some HIV-infected children charts and similar articles, a standardized data extraction checklist was carefully developed. The resulting data extraction tool had the following components: sociodemographic characteristics of caregivers and their children and clinical characteristics and nutritional status of the children. Trained data collectors carefully extracted all available data from the selected medical records of HIV-positive children. The most recent laboratory and clinical measurements documented at ART initiation were used as baseline values. A daily base supervision was done by assigned supervisors to maintain data quality. Besides, training concerning data extraction process was given for both data collectors and supervisors. Lastly, the consistency between records and collected data was confirmed by principal investigators through randomly selected reviews of previously extracted medical records.

### Measurements

The death of any HIV-positive child after ART initiation during the five follow-up period was considered as an event. Conversely, study participants who were still alive at the end of follow-up, lost from the study, or transferred to other health institutions were considered as censored.

ART drug adherence level was calculated based on the total monthly doses of ART. Good ART adherence was documented when the compliance level was ≥95% or missed doses were ≤ three per month. Fair ART adherence was documented when the compliance level was between 85 and 94% or missed doses were between four and eight per month. Lastly, poor ART adherence was documented when the compliance level was < 85% or missed doses were ≥ nine per month [13].

HIV-positive children with weight for age (W/Age) Z-score < − 2 SD, height for age (H/Age) Z-score < − 2 SD, and weight for height (W/H) Z-score < − 2 SD were classified as moderately underweight, stunted, and wasted respectively [14, 15].

HIV-positive children with W/Age Z-score < − 3 SD, H/Age Z-score < − 3 SD, and W/H Z-score < − 3 SD were classified as severely underweight, stunted, and wasted respectively [14, 15].

The CD4 count below the threshold for ages < 12 months, 12–35 months, 36–59 months, and ≥ 5 years were < 1500/mm^3^, < 750/mm^3^, < 350/mm^3^, and < 200/mm^3^ respectively [16].

### Data management and statistical analysis

Epi-Data™ Version 3.1 was used for data entry and STATA™ Version 14 was used for statistical analysis. Descriptive statistics for continuous variables were summarized using mean, median, and standard deviation. The survival probability among HIV-infected children from the date of ART initiation to event was estimated using the Kaplan Meier survival curve. Cox proportional hazard regression model was fitted, then all variables with *p*-value ≤0.25 in the bivariable analysis were included in a multivariable model. Cox-proportional hazard model assumption was checked using Schoenfeld residual test for continuous variables and Log-Log plots for categorical variables. Besides, multicollinearity was checked using the variance inflation factor (VIF). In the final model, hazard ratios with 95% confidence intervals and *p*-values (< 0.05) were used to identify statistically significant predictors and measure the strength of association.

## Results

### Demographic characteristics of included participants

After reviewing 553 HIV-infected children’s records, 538 records were included in the final analysis as, 15 records were excluded due to incompleteness. About half (51.5%) of study participants were females and the majority of participants (83.6%) were from urban areas. The baseline mean age of the participant cohort was 6.7 years (SD: 3.8 years); with the mean age of caregivers reported as 33.1 years (SD: 0.4 year). Details of another captured socio-demographic characteristics are found in Table 1.

**Table 1:** Baseline demographic characteristics of HIV infected children receiving ART in Amhara Region Referral Hospitals between January 2012 and February 2017

Variables	Frequency (N)	Percentage (%)
**Sex**		
Male	261	48.5
Female	277	51.5
**Age**		
< 1 year	22	4.1
1–4 years	169	31.4
5–15 years	347	64.5
**Residence**		
Urban	450	83.6
Rural	88	16.4
**Caregiver marital status (525)**		
Single	58	11.1
Married	274	52.2
Divorced	98	18.7
Widowed	95	18.1
**Parent status**		
Both alive	323	60
Father dead	94	17
Mother dead	48	9
Both dead	73	14
**Caregiver of the child**		
Parent	452	84.0
Sister/Brother	19	3.5
Uncle/aunt	16	3.0
Grandparent	31	5.8
Others	20	3.7

### Baseline clinical, laboratory, and ART information

**Fig. 2:**
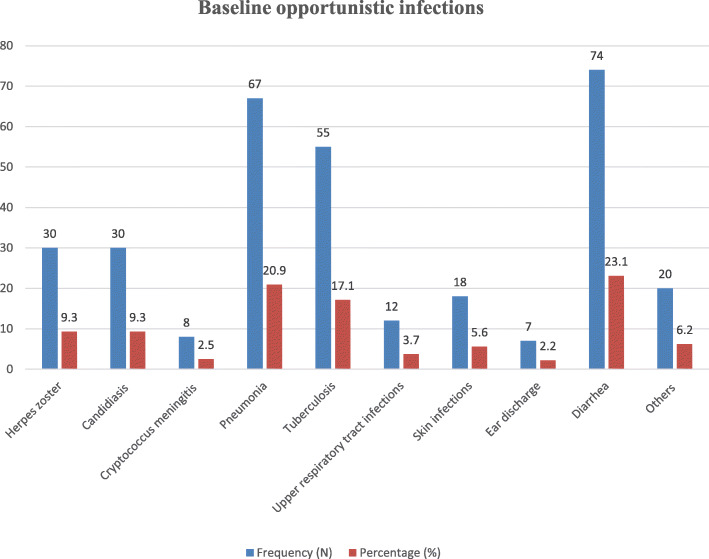
Baseline opportunistic infections of HIV-infected children receiving ART in Amhara Region Referral Hospitals from January 1, 2012 to February 28, 2017

About 45.0% of the HIV-positive children had opportunistic infections (OIs) at ART initiation. Of these, 23.3, 20.9, and 17.1% of the children had diarrhea, pneumonia, and tuberculosis, respectively (Fig. 2). About 57.6% of the HIV-positive children had mild WHO clinical disease staging (stage I and II). Moreover, nearly two-thirds (69.5%) of the children had CD4 counts below the threshold. Furthermore, less than one-third (30.8%) of the participants had anemia at ART initiation. Regarding baseline nutritional status, 17.3, 22.7, and 16.7% of HIV-infected children were severely underweight, stunted, and wasted. Furthermore, about 12.3% of the children were anemic during ART initiation (Table 2).

**Table 2:** Clinical, laboratory, and ART information of HIV infected children receiving ART in Amhara Region Referral Hospitals from January 1, 2012 to February 28, 2017

Variables	Frequency (N)	Percentage (%)
**OI at baseline**		
Yes	242	45.0
No	296	55.0
**Functional status (age** ***≥*** **5 years) (*N*** **= 347)**		
Working	195	56.2
Ambulatory	144	41.5
Bedridden	8	2.3
**Developmental history (age < 5 years) (*N*** **= 191)**		
Appropriate	143	74.9
Delayed	40	20.9
Regressive	8	4.2
**WHO clinical staging**		
Stage I and II	310	57.6
Stage III and IV	228	42.4
**Past TB test before ART**		
Not determined	430	80.0
Negative	53	9.9
Positive	55	10.1
**Past TB treatment**		
No treatment	483	89.8
2SRHZ/4RH	38	7.1
2HRZES/4HRE	4	0.8
HRZE/4RH	13	2.4
**CD4 count or percent**		
Severe immunodeficiency	374	69.5
Mild immunodeficiency	164	30.5
**Hemoglobin level**		
Anemic(< 10 g/dl)	66	12.3
Non-anemic (≥10 g/dl)	472	87.7
**ART eligibility criteria**		
Immunologic/ CD4	134	24.9
WHO clinical stage	67	12.5
Both clinical and immunologic	216	40.2
Test and treat approach	121	22.5
**Cotrimoxazole preventive therapy**		
Yes	340	63.2
No	198	36.8
**ART adherence in the 1st 3 months**		
Good /fair	495	92.2
Poor	42	7.8
**Underweight (baseline)**		
Normal	317	58.9
Moderate (WAZ < - 2)	128	23.8
Severe (WAZ < −3)	93	17.3
**Stunting (baseline)**		
Normal	336	62.5
Moderate (HAZ < −2)	80	14.9
Severe (HAZ < −3)	122	22.7
**Wasting (baseline)**		
Normal	365	67.8
Moderate (WHZ or BAZ < −2)	83	15.4
Severe (WHZ or BAZ < − 3)	90	16.7

### Follow-up characteristics of the children

**Fig. 3:**
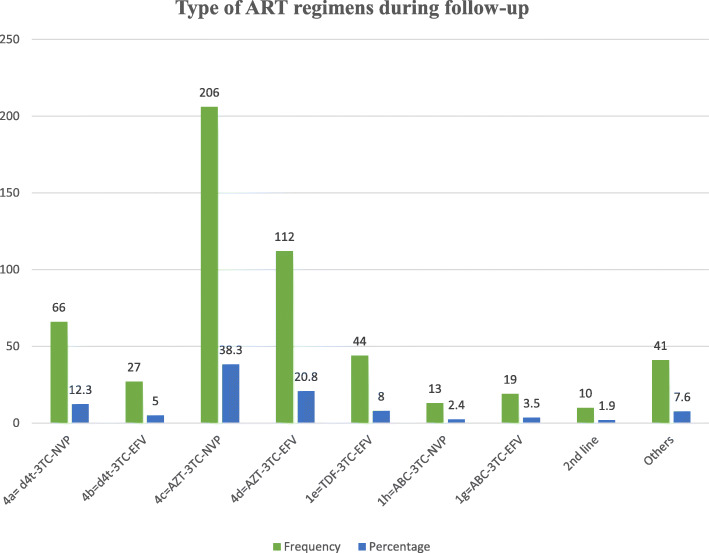
Type of regimens during follow-up among HIV-infected children receiving ART in Amhara Region Referral Hospitals from January 1, 2012 to February 28, 2017

Throughout the follow-up time, 17.8% of the study participants developed ART related side effects. Additionally, nearly one-fourth (26.4%) of the children developed OIs during the follow-up time. Furthermore, almost one-third (29.7%) of HIV-infected children had a history of ART regimen change during the follow-up time. Interestingly, about 4.7% of the children discontinued their medication during the follow-up time. More than half (59.1%) of the HIV-positive children were taking a combination of zidovudine, lamivudine, and efavirenz or zidovudine, lamivudine, and nevirapine (Fig. 3).

### Mortality rate during our follow-up

**Fig. 4:**
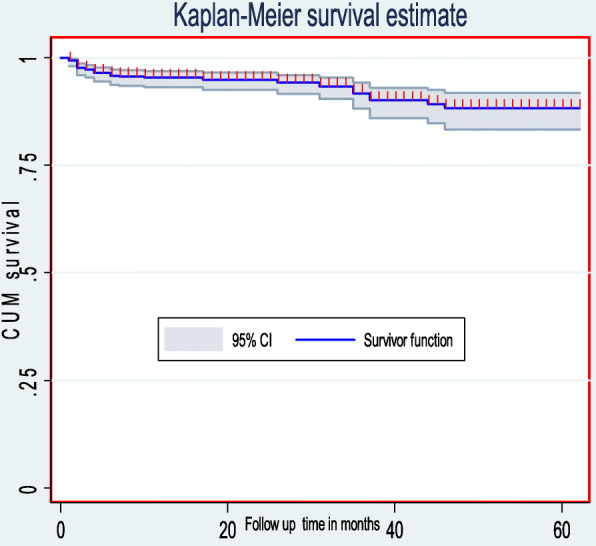
The overall Kaplan-Meier survival curve with 95% confidence intervals of HIV-infected children receiving ART in Amhara Region Referral Hospitals from January 1, 2012 to February 28, 2017

This cohort contributed a total follow-up time of 14,600 child-months. Among a cohort of 538 HIV-infected children, 38 children (7.1%) died, 33 (6.1%) were lost to follow-up, 81(15.1%) were transferred to other health institutions, and 386 (71.8%) remained in the follow-up. Finally, the combined mortality rate of this cohort was 3.2 (95% CI: 2.3, 4.3) per 100 child-years of observation. The number of deaths within the first three, six, and 12 months of ART follow-up were 19 (50%), 24 (63%), and 26 (68%), respectively. The 60 months cumulative survival probability of HIV-infected children was 0.88 (95% CI: 0.84, 0.92) (Fig. 4).

### Predictors of mortality

In the final model, the following factors increased the risk of mortality among HIV-positive children: baseline OIs, anemia, severe immunodeficiency, advanced disease stage, severe stunting, and severe wasting. We observed that the hazard of death among children presenting with OIs at ART initiation was 2.5 fold (AHR: 2.5**,** 95%CI: 1.04, 5.9) higher than to those presenting without OIs. Additionally, the hazard of death among children who initiated ART with severe disease was three-fold higher as compared to those who initiated ART with mild disease (AHR: 3.0, 95% CI: 1.2, 7.1). Furthermore, the hazard of death among children who had severe immunodeficiency was 4.4 fold (AHR: 4.4, 95% CI: 1.7, 11.7) higher than to those who had mild immunodeficiency. The hazard of death among anemic children was three-fold (AHR: 3.1, 95% CI: 1.4, 6.7) higher as compared to non-anemic children. Lastly, the hazard of death among severely stunted, and severely wasted children was three times higher as compared to their well-nourished counterparts [(AHR: 3.3, 95% CI: 1.4, 8.0), and (AHR: 3.1, 95% CI: 1.3, 7.3)] respectively (Table 3).

**Table 3:** The bivariable and multivariable Cox regression analysis of predictors of mortality among HIV infected children receiving ART in Amhara Region Referral Hospitals from January 1, 2012 to February 28, 2017

Variables	Survival status		CHR (95%CI)	AHR (95%CI)
	Dead	Censored		
**Sex**				
Male	26	235	2.3 (1.2, 4.5)	1.4 (0.7, 2.9)
Female	12	265	1	1
**Age**				
< 1 year	3	19	3.1 (0.9, 10.3)	2.8 (0.7, 10.7)
1–5 years	17	152	1.9 (1.0, 3.7)	1.6 (0.8, 3.5)
5–15 years	18	329	1	1
**Residence**				
Urban	35	415	1	1
Rural	3	85	0.5 (0.2, 1.6)	0.5 (0.1, 1.6)
**OI at baseline**				
Yes	30	212	4.6 (2.1, 10.0)	2.5 (1.04, 5.9)^a^
No	8	288	1	1
**WHO clinical staging**				
Stage I and II	8	302	1	1
Stage III and IV	30	198	5.3 (2.4, 11.5)	3.0 (1.2, 7.1)^a^
**CD4 count or percent**				
Severe immunodeficiency	33	341	3.0 (1.2, 7.7)	4.4 (1.7, 11.7)^a^
Mild immunodeficiency	5	159	1	1
**Anemia**				
Anemic (< 10 g/dl)	15	51	5.4 (2.8, 10.4)	3.1 (1.4, 6.7)^a^
Non-anemic (≥10 g/dl)	23	449	1	1
**Stunting**				
Normal	12	324	1	1
Moderate (HAZ < −2)	8	72	3.0 (1.2, 6.9)	2.0 (0.8, 5.3)
Severe (HAZ < −3)	18	104	4.6 (2.0, 9.5)	3.3 (1.4, 8.0)^a^
**Wasting**				
Normal	18	347	1	1
Moderate (WHZ or BAZ < −2)	5	78	1.1 (0.4, 3.0)	1.3 (0.4, 3.7)
Severe (WHZ or BAZ < −3)	15	75	3.2 (1.6, 6.5)	3.1 (1.3, 7.3)^a^

^**a**^ Significant predictors in the multivariable analysis

## Discussion

This study identified the predictors of mortality among HIV-positive children receiving ART using a multicenter facility-based historical cohort study. Within a total follow-up time of 14,600 child-months, the mortality rate of HIV-positive children receiving ART in our study hospitals was 3.2 (95% CI: 2.3, 4.3) per 100 child-years of observation. Our finding is comparable with studies reported from Arba Minch Town Public Health Facilities, Gamo Gofa Zone, Southern Ethiopia (3.1 deaths per 100 child-years) [17], Nigeria (3.0 deaths per 100 child-years) [18], and Andhra Pradesh, India (3.0 deaths per 100 child-years) [19].

Nonetheless, our finding is lower than studies conducted in Addis Ababa Public Hospitals, Ethiopia (12.4 deaths per 100 child-years) [20], Debre Tabor General Hospital and Dessie Referral Hospital, Ethiopia (6.3 deaths per 100 child-years) [21], and Kenya (8.4 deaths per 100 child-years) [22]. These significant disparities across the reported studies could be due to the differences in sample size, study setting, follow-up period, and clinical characteristics of the study participants. As an example, the follow-up period for a study done at Debre Tabor General Hospital and Dessie Referral Hospital was 120 months [21], whereas the follow-up period of our study was 60 months. It is well understood that as the follow-up period increases, the number of events (death) also increases. Additionally, a study conducted in Kenya included only 135 HIV-infected children [22]; however, our study included 538 HIV-infected children with the smaller sample size potentially yielding a less precise estimation.

This study also found a high rate of mortality when compared to studies reported from Adama Referral Hospital and Medical College, Ethiopia (2.1 deaths per 100 child-years) [23], Zambia (1.6 deaths per 100 child-years) [24], and Malawi, Lesotho, and Swaziland (2.25 deaths per 100 child-years) [25]. The above variations might be due to the differences in clinical characteristics of the study participants and study settings as our study included only referral hospitals. Additionally, our study’s higher rate of mortality could be attributed to the clinical characteristics of the included study participants such as the nearly two-thirds (69.5%) of our study participants having severe immunodeficiency.

Of 38 total deaths, 68% occurred within the first year of ART initiation. This finding is comparable with a study done at Bahir Dar Referral Hospital, Ethiopia, found that the mortality rate within the first year after ART commencement was 90.2% [5]. Moreover, this finding is consistent with studies done in other SSA countries, which documented that the death of most HIV-positive children commonly happened in the early phase of ART [26, 27]. Higher premature mortality in our study might be associated with the clinical characteristics of included participants as over two-thirds (69.5%) of the study participants had CD4 counts below the threshold, a population which usually experiences higher early mortality after ART initiation. Moreover, children with weakened immunity are more easily attacked by different types of OIs, which are the most common causes of premature death.

This study found that the risk of mortality among anemic HIV-positive children was three-fold higher as compared to their non-anemic counterparts. Previous Ethiopian studies also documented that anemia had a significant impact on the survival of HIV-infected children [5, 9, 10, 12]. Additionally, studies reported from other LMICs also reported that anemia at ART initiation was significantly associated with higher mortality [22, 27, 28]. Furthermore, studies conducted elsewhere also demonstrated that anemia is the most common hematologic abnormality among people living with HIV. It is significantly associated with low quality of life and a higher risk of mortality [29, 30]. The most common cause of anemia among people living with HIV is the side effects of zidovudine, which 59.1% of our study participants were taking a combination of Zidovudine, lamivudine, and efavirenz or Zidovudine, lamivudine, and nevirapine. Cotrimoxazole preventive therapy (CPT), being reflected in 63.2% of our participants, can be an additional cause of anemia among HIV-positive children.

This study also revealed that HIV-positive children presenting with OIs at ART initiation were at higher risk of mortality. A Tanzanian study also reported that children who had TB and other OIs at ART initiation were at higher risk of mortality [28]. Besides, a study from South Africa also demonstrated that OIs in the form of chronic diarrhea was a significant predictor of mortality [26]. As OIs are the predominant causes of morbidity and mortality among HIV-infected patients, the Ethiopian National ART Guidelines strongly recommends treating OIs before ART initiation [6]. In addition to the direct effect of these OIs, patients may have died due to the side effects of common drugs used to treat these OIs. Concerning drug-drug interactions, studies suggested that HIV-infected individuals treated with anti-TB medications usually experience drug toxicity as compared to HIV-uninfected individuals [31, 32]. As an example, a study done in Canada revealed that HIV-infected people treated with anti-TB medications were more likely to develop drug-related adverse events [32].

Further finding of this current study demonstrated that children who had severe immunodeficiency were at higher risk of death as compared to those who had mild immunodeficiency. This finding is in agreement with the previous Ethiopian studies [5, 9, 12, 21]. Besides, a Brazilian study also reported that baseline CD4 count below 15% significantly increased the hazard of death among HIV-positive children [33]. Similarly, studies conducted elsewhere have documented that HIV-positive children with low CD4 count were at higher risk of mortality than those who had high CD4 count [28, 34–36]. Generally, serious and life-threatening OIs, including CNS toxoplasmosis and cryptococcal meningitis are frequent among people having low CD4 count or percentages which increases their vulnerability to death.

Another finding of this study revealed that the death rate among children with advanced disease stage (WHO stage III and IV) was higher as compared to those children with mild disease stage (WHO stage I and II). This finding is highly supported by previous studies conducted elsewhere [9, 10, 22, 23, 37, 38]. OIs are the most common morbidities among HIV-positive children with advanced WHO clinical disease stage, which indicates that the risk of developing and recurrence of OIs increases as the WHO clinical staging becomes more advanced. Hence, people living with HIV commonly died due to OIs rather than HIV/AIDS.

Finally, this study found that undernutrition in the form severe stunting (H/Age Z-score < − 3 SD) and severe wasting (W/H Z-score < − 3 SD) significantly increased the risk of mortality among HIV-infected children on ART. This finding is comparable with the previous Ethiopian studies conducted elsewhere [11, 12]. Additionally, a Kenyan study also revealed that those children who had baseline WHZ (Weight for Age Z-score) < − 2 were at higher risk of death among HIV-positive children receiving HAART [22]. Moreover, a Tanzanian study also found that WHZ and BAZ (Body Mass Index for Age Z-score) of ≤ -2 significantly associated with higher risk mortality within the first 3 months of ART initiation [28]. Generally, the causes of undernutrition among HIV-patients are multidimensional and multifactorial. HIV decreases food consumption as a result of swallowing difficulty caused by oral trash or esophageal candidiasis, reduces nutritional absorption caused by gastrointestinal mucosal damage, and increases the metabolic demands [39, 40].

## Limitations of the study

The major limitation of this study was the lack of quality data to verify death status, especially for those who died at home. This gap does not only affect the loss to follow-up, but also may be an under-reporting of outcomes of community-based individuals. In addition, this study did not incorporate important predictors, like viral load and micronutrient deficiency due to incomplete recording system. Finally, early mortality could be underestimated since the study excluded children’s who had ART follow-up was less than 1 month.

## Conclusions

A higher rate of mortality was observed in our study as compared to previous Ethiopian studies. Anemia, OIs, low CD4 counts or percentages, advanced disease staging (III and IV), severe stunting, and severe wasting were factors found as significantly increasing the risk of mortality among HIV-infected children receiving ART. Therefore, clinicians should emphasize early screening, managing OIs, and maximizing nutritional supplements for HIV infected children to improve the survival rate of those individuals. Additionally, clinicians should give more emphasis to malnourishment in HIV-infected children at ART initiation. Furthermore, greater attention and close follow-up shall be given for HIV-infected children in the early ART phase.

## Data Availability

Data will be available upon request of the corresponding author.

## Abbreviations

AIDS: Acquired Immune Deficiency Syndrome
AZT- 3TC-EFV: Zidovudine plus Lamivudine plus Efavirenz
AZT-3TC-NVP: Zidovudine plus Lamivudine plus Nevirapine
BAZ: Body Mass Index for Age Z-score,
HAART: Highly Active Antiretroviral Therapy
HAZ: Height for Age Z-score
Hgb: Hemoglobin
HIV: Human Immunodeficiency Virus
SSA: Sub-Saharan Africa
WAZ: Weight for Age Z-score
WHO: World Health Organization
WHZ: Weight for Height Z score
